# HYPERVASCULAR LIVER LESIONS IN RADIOLOGICALLY NORMAL LIVER

**DOI:** 10.1590/0102-6720201700010007

**Published:** 2017

**Authors:** Enio Campos AMICO, José Roberto ALVES, Dyego Leandro Bezerra de SOUZA, Fellipe Alexandre Macena SALVIANO, Samir Assi JOÃO, Adriano de Araújo Lima LIGUORI

**Affiliations:** Gastrocentro Clinic and Clinic for Digestive Tract Surgery and Hepato-biliary-pancreatic Surgery, Onofre Lopes University Hospital, Federal University of Rio Grande do Norte, Natal, RN, Brazil.

**Keywords:** Diagnosis, Liver neoplasms, Focal nodular hyperplasia, Adenoma, Liver cell neoplasm, Metastasis

## Abstract

**Background:**

The hypervascular liver lesions represent a diagnostic challenge.

**Aim:**

To identify risk factors for cancer in patients with non-hemangiomatous hypervascular hepatic lesions in radiologically normal liver.

**Method:**

This prospective study included patients with hypervascular liver lesions in radiologically normal liver. The diagnosis was made by biopsy or was presumed on the basis of radiologic stability in follow-up period of one year. Cirrhosis or patients with typical imaging characteristics of haemangioma were excluded.

**Results:**

Eighty-eight patients were included. The average age was 42.4. The lesions were unique and were between 2-5 cm in size in most cases. Liver biopsy was performed in approximately 1/3 of cases. The lesions were benign or most likely benign in 81.8%, while cancer was diagnosed in 12.5% of cases. Univariate analysis showed that age >45 years (p< 0.001), personal history of cancer (p=0.020), presence of >3 nodules (p=0.003) and elevated alkaline phosphatase (p=0.013) were significant risk factors for cancer.

**Conclusion:**

It is safe to observe hypervascular liver lesions in normal liver in patients up to 45 years, normal alanine aminotransaminase, up to three nodules and no personal history of cancer. Lesion biopsies are safe in patients with atypical lesions and define the treatment to be established for most of these patients.

## RESUMO

**Racional:**

As lesões hepáticas hipervasculares representam um desafio diagnóstico.

**Objetivo:**

Identificar fatores de risco para câncer em pacientes portadores de lesão hepática hipervascular não-hemangiomatosa em fígado radiologicamente normal.

**Método:**

Estudo prospectivo que incluiu pacientes com lesões hepáticas hipervasculares em que o diagnóstico final foi obtido por exame anatomopatológico ou, presumido a partir de seguimento mínimo de um ano. Diagnóstico prévio de cirrose ou radiológico de hemangioma foram considerados critérios de exclusão.

**Resultados:**

Oitenta e oito pacientes foram incluídos. A relação mulher/homem foi de 5,3/1. A idade média foi de 42,4 anos. Na maior parte das vezes as lesões hepáticas foram únicas e com tamanho entre 2-5 cm. Em aproximadamente 1/3 dos casos foi realizada biópsia hepática. Em 81,8% dos casos as lesões eram benignas ou provavelmente benignas enquanto que em 12,5% dos casos o diagnóstico foi de câncer. A análise univariada mostrou que idade superior a 45 anos (p<0,001), antecedente familiar pessoal de câncer (p=0,020), presença de mais de três nódulos (p=0,003) e elevação da alanina aminotransaminase (p=0,013) foram fatores de risco relevantes para o câncer.

**Conclusões:**

È indicado observar lesões hepáticas hipervasculares em fígado normal em pacientes com até 45 anos, alanina aminotransaminase normal, com até três nódulos e sem antecedente pessoal de câncer. Para os demais com lesões atípicas, a biópsia da lesão é segura e define na maior parte dos pacientes o tratamento a ser instituído.

## INTRODUCTION

Solid liver lesions have always been a cause for concern in clinical practice due to the known capacity of the liver to accommodate primary tumors and extrahepatic metastases. However, malignant tumors are not the only possibility. Benign lesions are also very common, with a prevalence of up to 20% in necropsy studies^8^. Defining the benign or malignant nature of these lesions is crucial, as the specific actions for treating each type of lesion are completely different, ranging from liver resections^18^ ^,^ ^19^ ^,^ ^21^ ^,^ ^27^, chemotherapy or even only imaging follow-up without any therapeutic intervention. Unlike what occurs in cirrhotic liver, in which hepatocellular carcinoma is often found and for which the literature has well-established algorithms for diagnosis^7^ ^,^ ^13^, the best way to establish solid liver lesion diagnoses in patients with normal livers is still not clear.

The standard of intravenous contrast enhancement during computed tomography (CT) and magnetic resonance imaging (MRI) exams is a fundamental step for the diagnostic evaluation of solid liver lesion. While hypovascular liver lesions are easy to diagnose because, excluding cysts and perfusion disorders, they are mostly caused by metastasis^4^, the hypervascular standard represents a major challenge. Benign solid lesions, hepatocellular carcinomas and some specific types of metastases, such as neuroendocrine tumors, are typically enhanced in relation to the hepatic parenchyma after the infusion of intravenous contrast, and the overlapping of the morphological characteristics of these lesions is not unusual. Few publications have been devoted to the diagnosis of patients with hipervascular liver lesion (HLL) in normal liver^9^ ^,^ ^26^. 

This study aims to identify the risk factors for the diagnosis of cancer in a population of patients with HLL and to try to establish a population of patients with undefined lesions in which a conservative approach with periodic imaging exams may be appropriate.

## METHODS

This prospective cohort study was performed from May 2007 to October 2015 and included patients with solid liver lesion of at least 1 cm in diameter in their longest axis, with single or multiple lesions present that were hypervascular in the arterial phase in the CT or MRI of the liver. Patients were treated at one of two outpatient clinics: the Gastrocentro Clinic (Natal / RN, Brazil) and the Clinic for Digestive Tract Surgery and for Hepato-biliary-pancreatic Surgery of the Onofre Lopes University Hospital - Federal University of Rio Grande do Norte, Natal, RN, Brazil. The definitive diagnosis of the lesion was established by imaging exams in cases of typical lesions or by anatomopathological examination from biopsies or surgical resections. For those patients for whom diagnosis was not possible, the nature of the lesion was suggested from a minimum follow-up period of one year with a new imaging exam (liver CT or MRI).

The exclusion criteria were: 1) clinical, laboratory or image diagnosis of cirrhosis and 2) liver lesions with typical aspects of hemangioma - in other words, evidence of centripetal filling of the lesion in the dynamic phase of CT or MRI in the initial examination - at the time of the inclusion in the study.

The CT and MRI exams were performed with different devices, depending on the location where the patient was treated. Minimum criteria for the quality of the radiological examination for inclusion in the study were established. CT exams were acquired using Multislice Helical CT devices with 16 channels or more, with sections with thicknesses of <5 mm and with three post-contrast phases (arterial, portal and equilibrium). For MRI, the studies were performed with 1.5 T devices with 8-channel surface coils. The protocol for the study of focal liver lesions consisted of pre-contrast T1 sequences in phase and out of phase, T2 with fat saturation and respiratory synchronization, T2 without fat saturation and with respiratory synchronization, and b 600-800 diffusion. In addition, a dynamic study with three-dimensional sequence-weighted T1 with fat saturation before and after intravenous extracellular gadolinium contrast medium application through injection pump in the arterial, portal and equilibrium phases was performed. The hepatobiliary phase was added approximately 20 min after the beginning of the injection for cases in which gadoxetic acid (Gd-EOB-DTPA - Primovist*(r)*; Bayer Schering, Berlin, Germany) was used.

### Diagnostic approach

Regarding the diagnostic approach, the patients were divided into three groups. 

**FIGURE 1:**
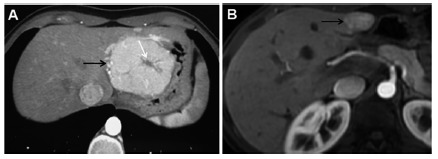
A) Arterial phase of a CT exam in a patient with typical FNH (black arrow) where a hypodense central scar can be observed in the center of the lesion (white arrow); B) arterial phases of MRIs in young patients with atypical arterialized lesions (white arrows) in steatotic liver where homogeneous emphasis of the lesions can be observed; control examinations at 24 months showed stability of the lesions, which were therefore considered "most likely benign"

1) The typical benign lesion group (G1): Patients with a typical diagnosis of either focal nodular hyperplasia (FNH) or hepatic adenoma (HA); a definitive diagnosis of FNH was made through the identification of a central scar, and a definitive diagnosis of HA was made when the lesion did not show whitening in the portal or equilibrium phase associated with the presence of intra- or perilesional hemorrhaging (Figure 1A). 

2) The immediate diagnosis group (G2): Patients who required an immediate diagnosis. The patients in this group were in one of two situations: G2.A, suspected malignancy (history of cancer with potential for liver metastasis; age ≥60 years, multiple lesions) or G2.B, lesions with dimensions ≥5 cm; the final diagnosis was required in this case because hepatic adenomas (HAs) with these dimensions have indications of resection^11^. 

3) The follow-up group (G3): Patients who were not in any of the previous groups; patients without a diagnosis but with little risk of malignancy and those who had suspected benign disease with lesions <5 cm were allocated on this group (Figure 1B).

### Therapeutic approach

The therapeutic approach was defined based on the group to which the patient belonged. 

**FIGURE 2:**
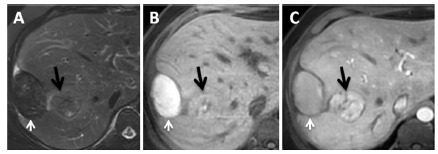
MRI in a patient with adenoma with bleeding: A) a T2 sequence with fat saturation shows a hepatic subcapsular nodule with hyposignal; B) a pre-contrast T1 sequence reveals a lesion with hypersignal, indicating products of hemoglobin degradation; C) a post-contrast T1 sequence in the arterial phase emphasizes the intra-hepatic lesion where the black arrow points to the lesion and the white arrow to the hematoma.

1) G1: Patients with FNH were subjected to a short follow-up with imaging exams unless they were symptomatic or in a situation in which surgery was indicated; once symptoms presented (i.e., bleeding), resection was indicated for the adenomas of this group, regardless of their size (Figure 2). 

2) G2: Patients in this group with suspected malignancies were investigated regarding tumor recurrence (in cases of being previous cancer patients) or were investigated for alleged primary tumors through a complete clinical evaluation, including upper gastrointestinal endoscopy, colonoscopy, thorax CT, chromogranin A and somatostatin analogue scintigraphy examinations (the last two examinations were performed preferably in the presence of multiple lesions). Biopsies were indicated for lesions in which the diagnosis was not defined with the cited exams. Each biopsy was performed with a 16 Gauge Tru-Cut needle through laparoscopic, laparotomic or percutaneous access. Based on the diagnosis found, the specific conduct was applied in each case. For patients in which diagnosis was not possible, a definitive diagnosis was suggested from the lesion behavior in the follow-up. 

3) G3: For patients in this group, clinical evaluations and follow-up examinations with CT or MRI were performed at 4 months, 8 months and then annually if the lesions were considered stable. Conventional MRI examination was preferably indicated in the patient follow-up because it is more accurate in the identification and characterization of liver lesions and because it is free of ionizing radiation. Biopsies were offered, when technically possible, for cases of increasing lesion size. Biopsies were also indicated in cases of differential diagnosis between HA and FNH <5 cm in which women expressed a desire to become pregnant or for patients who wished to definitely clarify the diagnosis at any time during the follow-up. For a few cases in this group and only in the last two years of the study, MRI with hepatic-specific contrast was performed as an option before an indication for biopsy. The diagnosis was considered typical of FNH in such cases when there was hyper/iso-signal in the hepatobiliary phase of the examination. The diagnosis was not considered typical if the hyposignal was identified in this stage of examination, as several types of lesions can occur with this finding.

#### Definition of lesions that underwent follow-up

A final diagnosis was suggested from the comparison between the size of the largest lesion in subsequent examinations for all patients in G3 and for some in G2 without clarifying the nature of the lesion. Thus, lesions that disappeared or decreased in size were considered "benign"; lesions that remained stable for two or more years were named "probably benign"; lesions that remained stable for a period between one and two years were considered "undefined"; and lesions that increased in size were defined as "suspected malignant".

### Statistical analysis

The mean and standard deviation were calculated for the quantitative variable age in the statistical analysis. The hypothesis test for differences between the groups in the dependent variable (benign or malignant lesion) was performed using the Student t test for independent samples at a 95% confidence level. The quantitative variables number of lesions and lesion size were categorized, and, along with the other categorical variables gender (male and female), use of contraceptives (yes or no), history of cancer (yes or no), symptoms (symptomatic and asymptomatic) and biopsy performed (yes or no), were analyzed using Pearson's chi-square or Fisher's exact tests. The magnitude of association was measured based on the relative risk (RR), and the confidence intervals were calculated at a 95% confidence level.

## RESULTS

One hundred and fifty-two patients with HLL were assessed during the study period. Forty-two patients were diagnosed with typical hemangioma and were excluded. Another 23 patients were not classified in any of the three study groups, although they had non-hemangiomatous HLL. The main cause of exclusion was a follow-up of less than one year. Thus, eighty-eight patients were included in the study. The patient and lesion characteristics are listed in Table 1. 

**TABLE 1:** Clinical characteristics of patients, the liver nodules and liver biopsy indications

	Groups			
	G1	G2	G3	Total
Clinical characteristics of patients				
Gender, female/male	18/3	32/6	24/5	74/14
Age, years, mean	31.2	51.4	38.5	42.4
Clinical presentation				
Asymptomatic	13	28	22	63 (71.6%)
Non-specific symptoms	5	6	7	18 (20.4%)
Related symptoms	3	4	0	7 (7.9%)
Personal history of cancer	0	20	0	19 (22.7%)
Characteristics of the nodules				
Number of lesions				
1	18	23	20	61 (69.3%)
2	2	0	8	10 (11.4%)
3	0	4	1	5 (5.7%)
> ou =4	1	11	0	12 (13.6%)
Diameter of the largest nodule				
1,0 - 1,5	0	7	6	13 (14.8%)
> 1,5 - 2,0	1	4	7	12 (13.6%)
> 2,0 - 5,0	8	18	16	42 (47.7%)
> 5,0	12	9	0	21 (23.9%)
Liver biopsy				
Indicated and performed	2	20	7	29 (32.9%)
Indicated and refused	0	5	0	5 (5.7%)
Inaccessible	0	6	0	6 (6.8%)
Not indicated	19	7	22	48 (54.5%)
Hepatectomy				
Immediate	4	3	0	7 (7.9%)
After the diagnosis	-	6	1	7 (7.9%)
TOTAL	21	38	29	88

The female/male ratio was 5.3/1. The average age of patients was 42.4 years. G1 had the lowest mean age (31.2 years), while G2 had the highest mean age (51.4 years). Asymptomatic lesions (incidentalomas) were characterized in 71.6% of cases. A personal history of cancer was present in 21.5% of cases. Regarding the characteristics of the nodules, in 69.3% of cases, the lesion was single, and in 71.6% of cases, the largest lesion measured >2 cm. Biopsy diagnoses were performed in approximately 1/3 of the patients and were indicated more often in G2. Hepatectomy was indicated in 14 cases due to suspected malignancy or to treat benign disease.

### GROUP 1 (G1)

Twenty-one patients were part of this group. Eighteen patients had FNH, and three patients had HA. Biopsy was indicated in two patients with typical aspects of FNH and who had large lesions (9.5 and 11 cm) in proximity to vascular structures. (Figures 1 and 2).

A patient with FNH (two lesions) underwent segmentectomy II/III + VII due to the presence of persistent abdominal pain. All patients with HA underwent hepatectomy. The surgical procedures were segmentectomy V, left hepatectomy and right hepatectomy + segmentectomy IVa. The patient who underwent left hepatectomy had a lesion 5.0 cm in diameter with recent bleeding undergoing surgery in the second trimester of pregnancy. Anatomopathological examination confirmed HA in all surgical cases.

### GROUP 2 (G2)

Thirty-eight patients were included in this group. Personal history of cancer was the most common risk factor found (n=19), with breast ductal carcinoma being the most frequent (n=4), followed by neuroendocrine tumor and colorectal adenocarcinoma (n=3 each). Fifteen patients were 60 years or older, eleven patients had multiple lesions (≥4) and eight patients had lesions >5 cm in the larger cross diameter.

Immediate diagnosis was obtained through biopsy (n=20), anatomopathological examination of the surgical sample (n=3) and positive testing for neuroendocrine tumor in OctreoScan (n=2), for a total of 25 patients. The preferred approaches for the 20 biopsies that were indicated and performed biopsies were laparoscopy (n=8), laparotomy (n=7) and percutaneous access (n=5). In most cases, the laparotomy indication was due to an associated surgical procedure.

Although biopsies were indicated for 11 patients, they were not performed due to either patient refusal or lesion inaccessibility. An imaging follow-up similar to that performed in G3 was conducted in all of these cases. During this follow-up, the diagnosis was of benign disease in five cases, as the lesions decreased in four cases and disappeared in one case. In the six other cases, the lesions were most classified as likely benign (n=2), undefined (n=2) and suspicious (n=2). The two patients with suspicious lesions refused biopsies based on negligible increases in size (3 mm in both cases) over a long period of follow-up (29 and 48 months).

MRI with gadoxetic acid was indicated for two patients (one was in the preoperative period for lobectomy for lung tumor) as a step prior to the indication of biopsy; examinations showed that both were cases of FNH.

The final diagnoses are shown in Table 2. 

**TABLE 2:** Final diagnoses of 88 LHHs

	Groups			
Diagnosis	G1	G2	G3	Total
Benign lesions	21	22	23	66 (75%)
FNH	18	8	9	35
HA	3	6	1	10
Hemangioma	-	3	1	4
Granuloma	-	-	1	1
Preserved area of parenchyma	-	-	1	1
Lesion disappeared	-	1	-	1
Lesion decreased	-	4	10	14
Most likely benign lesions*	-	2	4	6 (6.8%)
Undefined lesions**		2	1	3 (3.4%)
Suspicious lesions***	-	2	-	2 (2.3%)
Cancer	-	10	1	11 (12.5%)
Neuroendocrine tumor metastasis		4	1	5
Hepatocellular carcinoma		4	-	4
Colon metastatic adenocarcinoma		1	-	1
Gallbladder metastatic adenocarcinoma		1	-	1
Total	21 (23.9%)	38 (43.2%)	29 (32.9%)	88 (100%)

*Stable for two or more years. **Stable for <2 years. ***Increase.

As expected, this group contained more patients with cancer (n=10). The tumor types with the highest incidence rates were hepatocellular carcinoma and liver metastases from neuroendocrine tumors.

### GROUP 3 (G3)

Twenty-nine patients were included. The follow-up time ranged between 12 and 73 months. Biopsies of liver lesions were performed in seven cases. Six underwent MRI with gadoxetic acid. In four, the diagnosis was typical of FNH, including two patients whose lesions increased during follow-up. In a patient the lesion appeared hypointense relative to normal liver and atypical feature during hepatocellular phase was observed in another.

Twenty-three patients had benign lesions. The diagnosis of benign lesion was made due to lesion reduction in the follow-up (n=10) through biopsy (n=7), through MRI with hepatic-specific contrast (n=4) and through conventional MRI (n=2).

**FIGURE 3:**
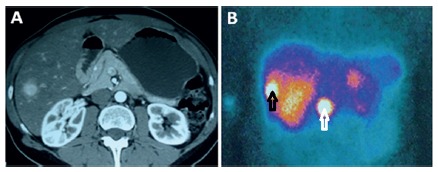
A) Arterial phase of a CT exam showing a homogeneous arterialized lesion in segment VI of the liver; B) OctreoScan examination revealed abnormal capture of the contrast in the pancreas (white arrow) and in the lesion previously identified on CT (black arrow). The diagnosis was pancreatic neuroendocrine tumor with liver metastasis.

The lesions were stable for over two years (n=4) or for less time (n=1), in five cases, although definitive diagnoses have not been obtained. The diagnosis was cancer in one case of a female patient 54 years of age with a 2-cm homogeneous arterialized lesion in the liver that remained stable in the first two years of follow-up. A pancreatic nodule and a 25% increase in a liver lesion were revealed via CT in the third year of follow-up. This patient underwent scintigraphy with an analogue of somatostatin (OctreoScan), which diagnosed neuroendocrine pancreatic tumor with liver metastases (Figure 3). 

The patient underwent pancreatoduodenectomy and resection of the VI segment of the liver, progressing uneventfully. A new suspicious hepatic nodule was evidenced in the follow-up and underwent percutaneous treatment with ablation.

### Cancer risk factors

The statistical analysis indicated that age >45 years (p<0.01), personal history of cancer (p=0.020), the presence of >3 nodules (p=0.003) and elevated alanine aminotransaminase (ALT) (p=0.013) were significant risk factors for cancer. Other factors such as gender (p=0,084), presence of symptoms (p=0,297) and lesion size (>5 cm x 1-5 cm; p=0,729/ >1,5 cm x 1-1,5 cm; p=0,657) were not related to cancer. When the patients were stratified between those who did not have any risk factors, regardless of the group to which they belonged (n=43) and those who had at least one risk factor (n=45), the cancer incidence rates were 0% and 24.4%, respectively (Table 3).

**TABLE 3:** Distribution of risk factors for cancer and occurrence of malignant liver lesions (cancer), benign and most likely benign

Risk factor	Cancer		Benign or most likely benign lesions		
	n	%	n	%	p
Age					
< or=45 year	0	0.0	50	100.0	<0.01*
> 45 years	11	33.3	22	66.6	
Gender					
Male	4	28.6	10	71.4	0.084
Female	7	10.1	62	89.9	
History of cancer					
Yes	6	30	14	70	0.020*
No	5	7.9	58	92.1	
ALPa					
Increased	5	35.7	9	64.3	0.013*
Normal	4	7.1	52	92.9	
Symptoms					
Yes	5	20	20	80	0.297
No	6	510.5	51	89.5	
Number of lesions					
4 or more	5	50	5	50	0.003*
Up to 3	5	8.2	67	91.8	
Size					
> 5 cm	2	9.5	19	90.5	0.729
1 - 5 cm	9	14.5	53	85.5	
> 1.5 cm	9	12.7	62	87.3	0.657
1 - 1.5 cm	2	16.7	10	83.3	

*Alanine aminotransaminase

## DISCUSSION

HLL have been studied since the introduction of the helical CT and sequences with rapid acquisition of magnetic nuclear resonance. The wide spectrum of diagnostic possibilities for HLL includes hemangiomas, focal nodular hyperplasias, hepatic adenomas, hepatocellular carcinoma and metastases from neuroendocrine tumors, among other lesions. The characteristics of these lesions in the dynamic phases of CT and MRI can safely establish noninvasive diagnoses for typical HLL. The problem is that in a significant number of cases, the characteristics of benign and malignant lesions overlap, with the same occurring among benign lesions. Excluding hemangiomas, which are easily diagnosed in most cases, diagnoses for other lesions may not be so obvious. Some examples can be described: 1) small adenomas are often mistaken for FNHs when they present fast homogeneous capture of contrast in the arterial phase of imaging^28^; 2) the central scar, considered a typical finding of FNH, is absent in a significant number of cases^16^; and 3) the washout found in the portal or equilibrium phases in CT or MRI is not an exclusive finding of hepatocellular carcinoma^14^, and it can be absent in this type of tumor^26^.

There is no clearly defined treatment for lesions that are considered undetermined in terms of malignancy. According to some authors, the best way to define the nature of the lesion is through a biopsy of a tissue fragment obtained from the lesion^25^. In addition to being technically difficult for small and deep lesions in the liver parenchyma, this invasive procedure may also be associated with risk of bleeding and tumor implantation in malignant lesions^11^. Follow-up with periodic imaging examination to evaluate changes in lesion size and characteristics is the other alternative for these cases. The biggest challenge involves defining which patients should be subjected to follow-up or should undergo biopsy.

In addition to the morphological characteristics identified in the imaging exams, the clinical scenario of patients with HLL appears to be relevant. This scenario is well recognized in the presence of liver cirrhosis in which hepatocellular carcinoma is very frequent and in patients with personal histories of cancer in which liver metastases are probable. According to the algorithm proposed by the American College of Radiology and recently updated in electronic publications^2^ ^,^ ^5^, patients with undetermined lesions and liver disease (cirrhosis, hepatitis B or C, primary sclerosing cholangitis and steatohepatitis) or history of malignant extrahepatic disease are classified as having high individual risk for malignant lesions. Extensive investigations through non-invasive exams or even liver biopsies in the persistence of doubt are recommended for those patients. Follow-up with imaging exams for undefined diagnoses is acknowledged as an alternative for the portion of patients without the aforementioned characteristics and, hence, low or medium risk of malignancy, although biopsy is also recommended. However, the patient population in which follow-up is recommended has not been specified.

This study's patient sample was composed of heterogeneous patients of both genders and various ages, symptomatic or not and sometimes with personal history of cancer. Nodule number and size were also variable. The overall incidence of cancer was 12.5%, when patients with a personal history of cancer were excluded from the study population, the incidence was 7.3%. While seemingly small, this incidence corresponds to a significant risk of cancer in a population made of potential candidates for conservative approaches, indicating that a previous diagnosis of cirrhosis and personal history of cancer are not the only significant risk factors to be considered. Age >45 years, elevated alanine aminotransferase and a number of nodules >3 cm are also significant risk factors for HLL malignancy. The stratification of a population of patients with minimal risk of cancer in which follow-up with imaging exams might be appropriate was made possible through these data. The data from this study cannot be compared with those of the medical literature, as no previous study has evaluated risk factors for cancer in patients with HLL; however, some series published on patients with liver incidentaloma have named advanced age, male gender, elevated alkaline phosphatase, and tumors larger than 4 cm, among others, as risk factors for cancer^4^ ^,^ ^17^ ^,^ ^26^. Interestingly, the inclusion of patients with chronic hepatitis or even cirrhosis, mainly in Asians, is casuistically frequent in these publications, which explains the high incidence of hepatocellular carcinoma in these series^26^. As stated above, the comparison between patients with HLL and incidentaloma does not seem appropriate for the desired analysis.

There are few studies that include series of patients with HLL; as mentioned, none of these studies analyze the risk factors for cancer in this specific population. The biggest study was a European multicenter investigation that retrospectively evaluated 550 patients with 910 HLL lesions^26^. The aim of the study was to evaluate the diagnostic accuracy of hepatic-specific contrast used in MRI exams. Because of the inclusion of cirrhotic patients, the incidence of hepatocellular carcinoma was 40.8% in the population studied. Because the study did not make the patient number explicit, it did not allow conclusions regarding the risk or even the incidence of cancer in patients with normal livers. In a recent publication, Chun et al. studied 79 patients with HLL in non-cirrhotic liver that were subjected to follow-up with imaging exams^9^. Only five patients had personal histories of cancer, only 14% were subjected to some type of intervention (embolization or surgery), and none of the patients had a diagnosis of cancer during the follow-up. Of the patients subjected to follow-up, 94% had stable lesions or even lesions that decreased in size. The major criticism for the study was the lack of a minimum follow-up time, i.e., patients were included in the study with no control imaging examinations. For patients with undetermined lesions, i.e., atypical lesions that were not biopsied or resected, a minimum follow-up period of one year or even longer is indispensable understand a lesion's behavior^9^. In the present study, this approach was followed to the extent in which only stable lesions in the 2-year period were considered as "most likely benign"; for lesions to be defined as "benign" by the proposed definition, a typical diagnosis through imaging examination, anatomopathological confirmation, size reduction or even lesion disappearance during the follow-up were necessary.

Although the need to biopsy liver lesions is low with modern imaging exams, the procedure is still frequently performed at various centers and is likely to increase in the coming years on suspicion of HA for better studies by immunohistochemistry^3^. Due to the use of stringent criteria for the diagnosis of typical benign lesions in this study, there was a high rate of biopsies performed (1/3 of cases). Bleeding, tumor implantation or equivocal results regarding the malignant or benign nature of lesions did not occur in any of the cases of this study as a result of the procedure. Whenever possible, due to the arterial nature of the lesions, the choice of this study was biopsy through laparoscopic access, which was performed in half of the cases. Percutaneous access was the preferred procedure for intraparenchymal lesions. The procedure needed to be repeated in only one case due to an insufficient amount of sample. The indication for immunohistochemistry was particularly useful when there was a diagnostic doubt between HA and FNH. This method was capable of diagnosing the nature of the lesion in all cases (n=14) in the present study. Bioulac-Sage et al. reported a substantial gain in diagnostic accuracy in this scenario when immunohistochemistry was added to conventional histology^6^. 

Perhaps the biggest criticism of the study is the small number of patients included in G1; in other words, in the group in which the diagnosis of typical benign lesion was possible through a non-invasive procedure with CT or MRI. In fact, only 29.2% of patients with benign or most likely benign lesions were included in this group. This small patient number was due to several factors: 1) most of the patients had been referred and had already received CT exams, which explains the small number of MRI performed as the initial examination in the casuistry; this situation contrasts with the recommendations of many authors, who judge the MRI as the exam of choice in the diagnostic evaluation of focal liver lesions^2^ ^,^ ^10^ ^,^ ^12^ ^,^ ^15^ ^,^ ^28^ ^,^ ^25^; the MRI performed in the follow-up examinations in the present study were able to clarify the diagnoses in at least six patients allocated into groups 2 and 3 who had not been diagnosed through CT and who had small hemangiomas, preserved areas of parenchyma in fatty liver and FNH; 2) a significant portion of patients (28.4%) had small lesions between 1 and 2 cm in size; the central scar of FNH and the heterogeneous areas of necrosis or hemorrhage of HA are infrequent in small lesions, which hampers the differential diagnosis of such lesions; 3) the definition of HA was based on rigid criteria, only including cases in which intra- or perilesional hemorrhaging was present; this feature was chosen because bleeding, although uncommon (21 - 40% of cases)^1^, is a reliable finding in the differentiation of FNH; 4) MRI with hepatic-specific contrast was not part of the early stage of the study, having been used in only a few cases in the last two years; the use of hepatic-specific contrast in MRI has been considered as the best way to establish a differential diagnosis between HA and FNH. In a recent meta-analysis that included 10 studies and 304 patients with FNH subjected to MRI with gadoxetic acid (Gd-EOB-DTPA), Suh et al^26^ concluded that High/Iso signal intensity on the hepatobiliary phase of the examination occurs in most patients (94-97%) with FNH. This finding has a highly accurate differential diagnosis with HA, which may be useful in avoiding unnecessary biopsy. This finding was observed in six patients in the present study who were subjected to examination and who, due the typical FNH diagnosis, were not biopsied. If MRI with gadoxetic acid had been performed routinely, it is likely that a large percentage of the ten patients belonging to groups 2 and 3 who had histopathological diagnoses of FNH would not have been subjected to biopsy, a fact that could reduce the indication for such a procedure for up to 1/3 of cases.

The biopsy may be unnecessary in many patients with the incorporation of magnetic resonance imaging with hepatic-specific contrast, which accurately diagnoses small focal nodular hyperplasias.

## CONCLUSION

At least 81.8% of the cases in a heterogeneous population of patients with HLL at least 1 cm in diameter are benign or most likely benign; FNH and HA are the most common lesions. HLL can be observed in normal liver in patients up to 45 years of age with a normal alanine aminotransaminase level and with up to three nodules identified on conventional CT or MRI. Lesion biopsy is safe in patients with atypical lesions and defines the treatment to be established for most of these patients. 
